# Combination of metformin and phenformin synergistically inhibits proliferation and hTERT expression in human breast cancer cells

**DOI:** 10.22038/IJBMS.2018.30460.7345

**Published:** 2018-11

**Authors:** Davoud Jafari-Gharabaghlou, Younes Pilehvar-Soltanahmadi, Mehdi Dadashpour, Ali Mota, Soheila Vafajouy-Jamshidi, Leila Faramarzi, Sara Rasouli, Nosratollah Zarghami

**Affiliations:** 1 Hematology and Oncology Research Center, Tabriz University of Medical Sciences, Tabriz, Iran; 2 Department of Clinical Biochemistry and Laboratory Medicine, Faculty of Medicine, Tabriz University of Medical Sciences, Tabriz, Iran; 3 Department of Medical Biotechnology, Faculty of Advanced Medical Sciences, Tabriz University of Medical Sciences, Tabriz, Iran; 4 Stem Cell Research Center, Tabriz University of Medical Sciences, Tabriz, Iran

**Keywords:** Breast cancer, Combination therapy, Metformin, Phenformin, Synergistic effects

## Abstract

**Objective(s):**

Breast cancer remains a global challenge, and further chemopreventive therapies are still immediately required. Emerging evidence has revealed the potent anti-cancer effects of biguanides, Metformin (MET) and phenformin (PHE). Thus, to explore an efficient chemopreventive strategy for breast cancer, the antiproliferative effects of the combination of MET and PHE against breast cancer cells were assessed.

**Materials and Methods:**

Cytotoxicity of the drugs individually and in combination against T47D and MDA-MB-231 breast cancer cells were assessed using MTT assay and the median-effect method was used to analyze the precise nature of the interaction between MET and PHE. Besides, the expression levels of hTERT after 48 hr drug exposure were determined using qRT-PCR.

**Results:**

Based on the cytotoxicity assay, both MET and PHE further inhibited the growth of MDA-MB-231 cells compared with T47D cells. It was found that MET+PHE reduced the IC50s of MET and PHE in both cells drastically more than the single treatments in a synergistic manner. Importantly, MET+PHE showed higher antiproliferative effect with smaller IC50 values against MDA-MB-231 cells than against T47D cells.

Real-time PCR results revealed that hTERT expression was significantly reduced in both breast cancer cell lines treated with MET+PHE than the single treatments. In comparison between two types of breast cancer cells, it was detected that MET+PHE could further decline hTERT expression in MDA-MB-231cells than in T47D cells (*P*<0.001).

**Conclusion:**

It is speculated that the combination of MET and PHE may be a promising and convenient approach to improve the efficiency of breast cancer treatment.speculated that the combination of MET and PHE may be a promising and convenient approach to improve the efficiency of breast cancer treatment.

## Introduction

Currently, breast cancer is the most common cancer among women in the U.S. and around the world due to its exceedingly high incidence (1, 2). In the U.S, about 1 in 8 women have been affected by breast cancer. In the United States, approximately 252,710 new cases of invasive breast cancer are expected to be detected in women in 2017, along with 63,410 new cases of *in situ* breast cancer. Moreover, about 40,610 women are estimated to die from the cancer (3).

 Though numerous chemotherapeutics such as paclitaxel, doxorubicin, and etoposide have been used to treat this type of cancer, issues such as low survival rates and high reoccurrence after conventional chemotherapy and radiation therapy remain. Thus, new targets and approaches should be developed (4). 

**Figure 1:**
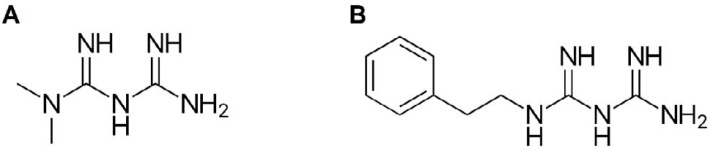
Chemical structures of (A) metformin and (B) phenformin

Several recent preclinical and clinical data suggest that the major biguanides, metformin (MET) and phenformin (PHE) (structures shown in Figure 1) with known pharmacokinetics, high safety profiles, and relatively low cost might be effective against various types of cancer including breast cancer (5). 

Combining two or more therapeutic agents, combination therapy, is a foundation stone in cancer treatment. The combination of chemotherapeutic agents enhances effectiveness compared to the mono-therapy strategy since it targets crucial pathways in a characteristically additive or synergistic manner (6, 7). This strategy potentially decreases drug resistance and concurrently supplies therapeutic anti-cancer advantages such as decreasing cancer cell proliferation and metastatic capacity, blocking mitotically active cells, decreasing the population of cancer stem cells, and stimulating apoptosis induction (8, 9).

The insulin-mediated systemic effects of MET lead to growth inhibitory effects against cancer cells. On the other hand, MET can inhibit the protein synthesis and cancer cell proliferation through modulation of the vital AMPK/mTOR/p70S6K pathway (10). Furthermore, activation of AMPK by MET results in the p53 phosphorylation, down-regulation of EGFR, cell cycle arrest, apoptosis induction, inhibition of activated ERK1/2, and autophagy (11). Both MET and PHE are biguanides with similar mechanisms of action (12). But the two drugs differ in potency. MET is functional in the liver, whereas PHE is able to get into cells easily and can affect many types of cells (5). 

Telomerase has been known as an attractive therapeutic target for treatment of different cancers, as it preserves tumor cell division and survival and decreases apoptosis induction (13-15). It has been shown that telomerase is active in 90% of breast carcinomas and 85% of human cancers, while in normal cells it is not active or detectable (16). Inhibition of telomerase activity especially its catalytic subunit, hTERT (human telomerase reverse transcriptase), in cancer cells may reactivate telomere shortening and might be a hopeful target in breast cancer treatment (17, 18).

Although MET and PHE have been revealed to display anti-cancer effects, the combination of both might show more efficient treatment of breast cancer. Therefore, in the present work, we took a step to survey the inhibitory effect of MET and PHE combination in the growth of T47D and MDA-MB-231 human breast cancer cell lines with a possible mechanism of telomerase inhibition. 

## Materials and Methods

***Chemicals and reagents***

Human breast cancer cell lines (T47D and MD-MB-231) were prepared from the cell bank of the Pasteur Institute of Iran. phenformin, metformin, streptomycin, and dimethyl sulfoxide (3- (4,5-Dimethylthiazol-2-yl)-2,5 diphenyltetrazolium bromide (MTT) powder were supplied by Sigma-Aldrich; RPMI 1640 and fetal bovine serum (FBS) were purchased from Gibco BRL (Life Technologies, Grand Island, NY, USA); sodium bicarbonate and penicillin G were purchased from Merck (Darmstadt, Germany); First Strand cDNA Synthesis Kit was purchased from Fermentas (Vilnius, Lithuania); and SYBR Green was purchased from Roche (Germany). All chemicals were used without further purification.

**Table 1:** Forward (F) and Reverse (R) primer sequences of hTERT and β-actin used in real-time PCR

Genes	Sequences	PCR product size (pb)
hTERT	F: 5′-CCCATTTCATCAGCAAGTTTGG-3′ R: 5′-CTTGGCTTTCAGGATGGAGTAG-3′	94
β-actin	F: 5′- GGTGAAGGTGACAGCAGT-3′ R: 5′- TGGGGTGGCTTTTAGGAT -3′	154

**Table 2:** IC_50_ and combination index (CI_50_) values for the drug formulations against T47D and MDA-MB-231 cells for 48 hr incubation time

Cell line	IC50 MET (mM)	IC50 PHE (mM)	IC50 of MET in combination (mM)	IC50 of PHE in combination (mM)	CI_50_
T47D	14.59	2.08	9.40	0.94	0.85
MDA-MB-231	11.35	1.08	4.01	0.40	0.63

**Figure 2:**
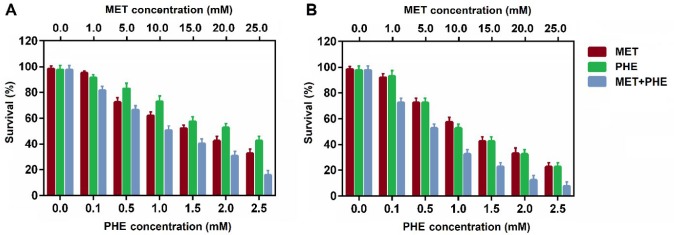
Effects of metformin, phenformin, and metformin+phenformin on breast cancer cell viability. (A) T47D and (B) MDA-MB-231 cells were treated with metformin, phenformin, and a combination of both drugs. Cell viability was measured using MTT assay after 48 hr treatment. Data represented are from three independent experiments

**Figure 3:**
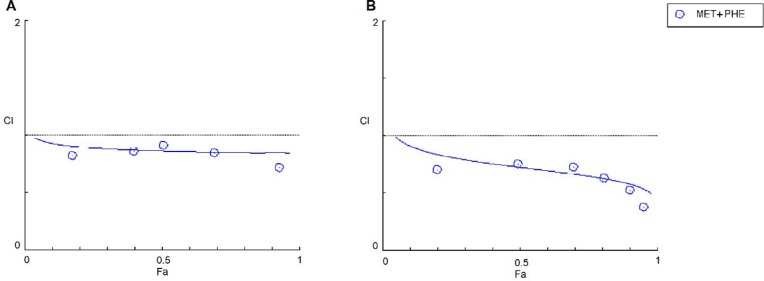
Synergistic inhibitory effects of metformin+phenformin on the growth of (A) T47D and (B) MDA-MB-231 breast cancer cells. Combination index (CI) was calculated by isobologram analysis using the Chou-Talalay method. CI =1, additive effect; CI <1, synergistic effect; CI >1, antagonistic effect. Data represented are from three independent experiments

**Figure 4:**
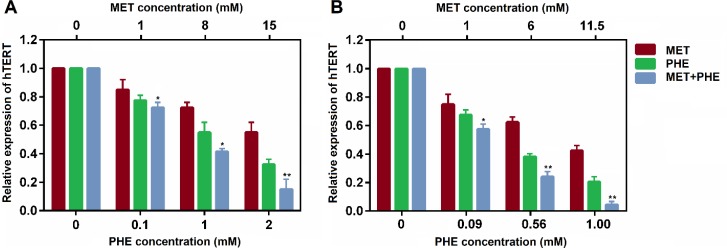
Inhibitory effects of metformin, phenformin, and metformin+phenformin on expression levels of hTERT in T47D and MDA-MB-231 breast cancer cells. *P< 0.05 and **P< 0.01 are the statistical differences between the combination form and individual drugs. Data represented are from three independent experiments

***In vitro*** **cytotoxicity**

RPMI 1640 medium supplemented with 10% FBS and 1% penicillin/streptomycin were used to culture T47D and MDA-MB-231 breast cancer cells. The cells were incubated in a humidified atmosphere containing 5% CO_2_ at 37 °C.

Cytotoxic activity was studied using MTT cell viability assay after 48 hr treatment of cells with MET, PHE, and their mixture. Metabolically active cells decrease the tetrazolium constituent of MTT to purple colored formazan crystals. Briefly, 2 × 10^4^ cells/well were cultivated in 96-well plates for 24 hr and then, treated with serial concentrations of MET (0, 1, 5, 10, 15, 20, and 25 mM), PHE (0, 0.1, 0.5, 1, 1.5, 2, and 2.5 mM) and MET+PHE. After 48 hr exposure time, the medium was replaced with fresh medium and 2 mg/ml of MTT was added to each well and plates were covered with aluminum foil and incubated for 4 hr at 37 °C. Thereafter, the content of the wells was removed and 200 μl pure DMSO and 25 μl Sorensen’s glycine buffer were added. In the next step, the absorbance of each well was read at 570 nm using ELISA plate reader (Bio-Tek Instruments) with reference wavelength of 630 nm. 

***RNA extraction, cDNA synthesis, and real-time PCR***

In this research, the mRNA level of the hTERT gene was studied, using real-time RT-PCR. T47D and MDA-MB-231 cells were treated with different concentrations of MET, PHE, and MET+PHE for 48 hr. After drug exposure time, total RNA was isolated using the Trizol reagent by referring to the manufacturer protocol. Then, the quantity and quality of total RNA were assessed based on OD260/280 ratio measurements and electrophoresis on 1.5% agarose, respectively. To gain cDNA, equal amounts of RNA were taken from all samples and reverse transcribed using RevertAid First Strand cDNA Synthesis Kit (Thermo scientific). Next, the level of hTERT gene expression was determined by the quantitative real-time PCR method using specific primers (Takapou Zist Co, Iran) (Table 1), and Hot Taq EvaGreen qPCR Mix was used according to the manufacturer’s protocol. The program for real-time PCR reaction was as follows; initial denaturation at 95 °C for 10 min, followed by cycles of denaturation at 95 °C for 15 sec, annealing at 60 °C for 30 sec, and extension at 72 °C for 30 sec. Finally, amplicons were measured by melting curve analysis of 70 °C to 95 °C. The real-time PCR efficiencies were determined for each gene. Relative hTERT expression levels were normalized by a housekeeping gene, β-actin, and calculated by this formula: normalized relative ratio *=*2 - *ΔΔ*Ct.

***Statistical analysis***

All experiments were done in three replicates and values displayed are representative for at least three independent experiments. Graph Pad Prism 6.7 was used for statistical analysis. All results of the experiments were expressed as the mean ± standard deviation (Mean ± SD). Levels of the statistical significance were measured using the paired Student t-test when comparing two groups, or by two-way ANOVA. *P*-values less than 0.05 were considered significant.

## Results

***Cytotoxicity and synergistic effect***

MTT assay was applied to assess the cytotoxic effects of MET, PHE and MET+PHE on T47D and MDA-MB-231 breast cancer cells after 48 hr treatment. The results showed that both MET and PHE significantly inhibited T47D and MDA-MB-231 cell growth in a dose-dependent manner (Figure 2). 

Table 2 shows the IC50 and combination index (CI_50_) values for the drug formulations against MDA-MB-231 cells for 48 hr incubation time. Based on the data analysis of the cytotoxicity assay, the IC50s of both MET and PHE for MDA-MB-231 cells were lower than T47D cells, indicating enhanced sensitivity of the TN phenotype. Also, it was found that the combination of MET and PHE led to a drastic reduction in IC50s of MET and PHE in two cell types relative to the single treatments. According to the IC50 values, MET and PHE in combination form showed higher antiproliferative effect with smaller IC_50_ values against MDA-MB-231 cells relative to the T47D cells. 

The precise nature of the interaction between MET and PHE in combination form was further analyzed by the median-effect method, where the CI values higher than, equal to, or lower than 1 indicate antagonism, additivity, or synergism in drug combinations, respectively (19). According to the combination index plot, the CI50s of MET+PHE for T47D and MDA-MB-231 cells were determined to be 0.85 and 0.55, respectively, which confirmed that the combination of MET and PHE had a synergistic effect against the proliferation of the cells (Figure 3). However, MET+PHE combination showed better synergistic growth inhibitory effect against MDA-MB-231 compared to T47D cells.

***hTERT expression***

To further explore the mechanisms involved in MET and PHE combination-mediated inhibition of breast cancer cells, qRT-PCR was applied to measure the expression levels of the hTERT gene. Therefore, the expression levels of the gene were determined after 48 hr drug treatment of breast cancer cells. 

Real-time PCR results showed that various concentrations of MET and PHE inhibited hTERT gene expression in T47D and MDA-MB-231 cells in a dose-dependent manner. As shown in Figure 4, significant reduction of hTERT expression was found in both breast cancer cell lines treated with MET+PHE compared with the single treatments. In comparison between two types of breast cancer cells, it was detected that the combination of MET and PHE could further decline hTERT expression in MDA-MB-231cells compared with T47D cells (*P*<0.05).

## Discussion

In the current work, the cytotoxic effects of MET and PHE combination were assessed against T47D and MDA-MB-231 breast cancer cells. The investigation revealed high capability of the combination to decrease the proliferation of breast cancer cells through suppression of hTERT expression.

We found that MET and PHE single treatments displayed antiproliferative effects against both types of breast cancer cells in a dose-dependent manner. The findings are consistent with those from previous reports that revealed the inhibitory activity of MET and PHE on the growth of different types of breast cancer cell lines (20-22). 

In agreement with previous reports, it was found that PHE has higher cytotoxicity towards the breast cancer cells compared with MET. Orecchioni and colleagues found that both MET and PHE activated AMPK, inhibited Complex 1 of the respiratory chain, and induced apoptosis of breast cancer cells and human white adipose tissue (WAT) progenitors (5). In co-culture, the biguanides inhibited the production of several angiogenic proteins. Also, the biguanides inhibited the local and metastatic growth of triple negative and HER21 breast cancer in immune-competent and immune-deficient mice orthotopically injected with breast cancer. It was found that PHE was significantly more potent compared to MET against breast cancer models both *in vitro* and *in vivo*, evidently since MET needs an organic cation transporter (OCT) to penetrate cancer cells (12). 

As a therapeutic for diabetes, PHE application was withdrawn from clinical application in relatively few countries in the late 1970s, due to a higher incidence of lactic acidosis in patients with renal failure relative to MET treatment, however, it was recently reported that supplementation of PHE with 2-deoxyglucose may prevent the risk of lactic acidosis (23). Hence, considering cancer therapy would be absolutely different from its previous clinical application for diabetes, PHE might be re-inspected as a potential anti-cancer agent to prevent and treat various cancers. 

MDA-MB-231 is a triple-negative breast cancer (TNBC) cell line with aggressive features and resistant to several anti-cancer drugs. Furthermore, MDA-MB-231 cells are for p53 and tumor-suppressor kinase LKB1, which make the cells even more resistant to anti-cancer agents (24). LKB1 is a key upstream kinase of the energy-sensing enzyme AMPK. It is thought that MET in culture conditions with high concentrations of glucose stimulates AMPK through an LKB1-dependent mechanism, which blocks mTOR, causing a powerful inhibition of cell proliferation in several types of cancer cells specially non-TN breast cancer cells (21, 25, 26). However, it is possible that normoglycemia normoglycemic conditions may be involved in the level of inhibitory efficiency of MET against cancer cells, potentially even in LKB1-deficient cells. Zordoky and colleagues revealed that normoglycemic conditions sensitize the LKB1-deficient cells such as MDA-MB-231 cells to the antiproliferative effect of MET via an AMPK-dependent mechanism (27). In accordance with these results, our MTT findings presented that MDA-MB-231 cells were more sensitive to MET and PHE in free and combined forms relative to T47D cells in normoglycemic conditions. 

In this study, we evaluated whether combining MET with PHE has a greater cytotoxicity effect against T47D and MDA-MB-231 breast cancer cells than the single treatment of the drugs. Our results suggest that MET and PHE in combination form were strongly effective in killing the cancer cells. The combination of MET and PHE markedly decreased cell growth in two breast cancer cell types relative to the single treatments in a synergistic manner. 

The combination of MET with chemotherapeutic agents such as paclitaxel, carboplatin, epirubicin, doxorubicin, 5-FU, and cyclophosphamide have been extensively reported (28-30). Recent data showed that MET in combination with trastuzumab killed cancer stem cells and inactivated ErbB2/IGF-1R interactions in a synergistic manner via inhibiting Src kinase and/or PI3K/Akt pathway, causing overwhelming primary resistance to trastuzumab in HER2 positive breast cancer cells (31, 32). Researchers showed that MET reduced migration and invasion of cancer cells in tamoxifen-resistant breast cancer cells and in combination with tamoxifen synergistically inhibited proliferation of ER-positive breast cancer via Bax/Bcl-2 and AMPK/mTOR/p70S6K pathways (33, 34). It was found that the combination treatment of MET and paclitaxel arrested in the G2/M phase, increased apoptosis and reduced cell proliferation in MCF-7 breast cancer cells (35). 

In one of the few studies, PHE plus oxamate (LDH; lactate dehydrogenase inhibitor) was assessed against various cancer cell lines including breast cancer, tonsil epithelial cancer, melanoma, and colon cancer (36). It was found that PHE and oxamate have synergistic anti-tumor effects via concurrent inhibition of mitochondria complex I and LDH in the cytosol, following acceleration of the production of reactive oxygen species (ROS). Oxamate in combination with PHE repressed LDH activity and lactate production by cells, resulting in prevention of lactic acidosis, which is a main side effect of biguanides, and rapidly encouraged cancer cell death through reducing ATP production and overproduction of ROS. 

Remarkably, MET experiences a variety of molecular mechanisms when it merges with various anti-cancer agent drugs. Inhibition of HIF-1, p-gp, and MRP1 expression plays a major function and is the major mechanism of MET when combined with anti-metabolites. On the other hand, activation of the AMPK/mTOR pathway is the major mechanism of MET in combination with hormone modulating drugs (37). Additionally, the synergistic effect of MET in combination with cisplatin was exerted through induction of apoptotic mitochondria and nucleus (38). In contrast, down-regulation of cholesterol biosynthesis is considered the molecular base of the anticancer effect of MET and taxane combination (37). Therefore, an exact survey of molecular mechanisms of MET in combination with various anticancer agents is crucial to comprehend its synergistic inhibitory effects and aid for personalized administration.

To explore the mechanisms involved in MET and PHE combination-mediated inhibition on breast cancer cells, the expression levels of the hTERT gene were assessed and our data suggest that the synergistic cytotoxic activity of the combination involves simultaneous inhibition of hTERT expression. Based on a study by Cantrell *et. al.*, telomerase activity can be regulated through MET treatment in various cancer cells. It was shown that MET strongly inhibits proliferation of endometrial cancer cells in a dose-dependent manner. Moreover, MET caused hTERT reduction, G1 arrest, and apoptosis induction (39). 

In the present study, the alteration in hTERT expression levels was surveyed to explain the molecular mechanism of MET and PHE combination-mediated synergistic anticancer effects. Nevertheless, deep exploration is required to provide exact mechanisms involved in the synergistic anticancer effects of the combined MET and PHE. Furthermore, considering some hurdles such as poor water solubility and low cellular uptake, which limit the effectiveness of the drugs, using co-nanodelivery systems may improve their bioavailability and cause strong synergistic anticancer effects against breast cancer cells (40-42). 

## Conclusion

The present work revealed that the combination of MET and PHE synergistically exerts growth inhibitory effects against T47D and MDA-MB-231 breast cancer cells through down-regulation of hTERT expression levels*. *According to these results, it can be suggested that the combinatorial chemotherapy based on MET and PHE may be a rational and cost-effective strategy for breast cancer therapy. 
